# Sub-Saharan African Countries: what challenges for achieving universal access to health?

**DOI:** 10.1590/1518-8345.0000.5051

**Published:** 2026-07-31

**Authors:** Fernando Mitano, Aline Aparecida Monroe, Pedro Fredemir Palha

**Affiliations:** 1 Universidade Lúrio, Faculdade de Ciências de Saúde, Nampula, NPL, Mozambique.; 2 Universidade de São Paulo, Escola de Enfermagem de Ribeirão Preto, PAHO/WHO Collaborating Centre for Nursing Research Development, Ribeirão Preto, SP, Brazil.

**Figure:**
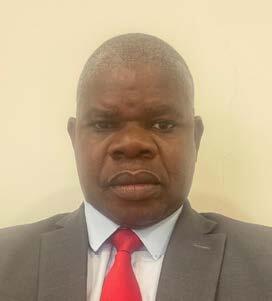

**Figure:**
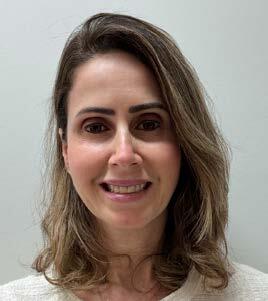

**Figure:**
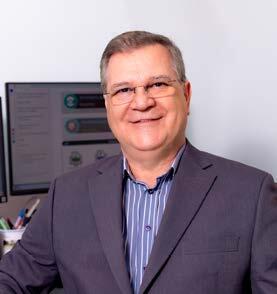

Quality health for all is a goal pursued by countries around the world, and it requires sustained efforts, both individually and collectively, from everyone, especially from the governments involved. In light of these conditions and the progress of the United Nations through the proposal of the Sustainable Development Goals (SDGs), efforts are made to overcome the structural deficiencies affecting populations living in low-, middle-, and high-income countries. The third of the seventeen SDGs imperatively advocates ensuring access to quality health care and promoting well-being for all people at all stages of life^1^. This goal is expected to be achieved by 2030; however, given the broad and deep structural inequalities, is this projection in fact applicable to Sub-Saharan African countries?

**Figure 1:**
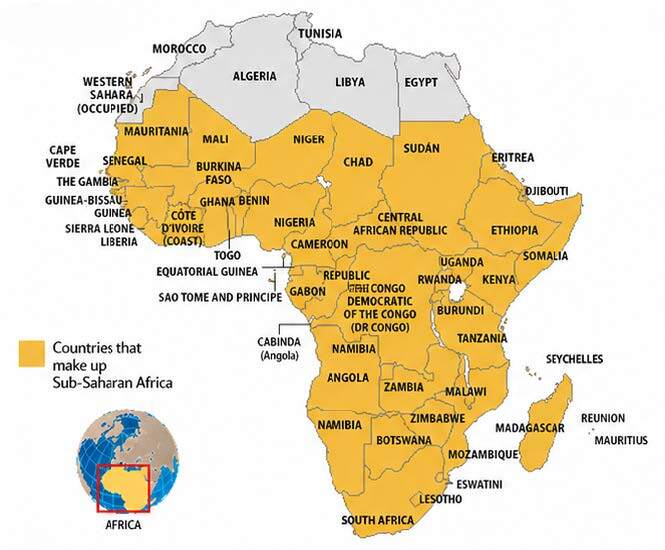
Sub-Saharan African Countries

Source: https://brasilescola.uol.com.br/geografia/as-duas-africas.htm

Despite global efforts to achieve this goal, Sub-Saharan Africa appears to be out of step with other regions of the world. This area, consisting of 47 countries located south of the Sahara Desert (Figure 1), has faced numerous health challenges whose mitigation requires political commitment, strategic management, and coordinated interventions aligned with the targets of SDG 3.

These targets include reducing the maternal mortality rate to less than 70 per 100,000 live births; eliminating preventable newborn deaths; ending epidemics of tuberculosis (TB), human immunodeficiency virus (HIV), malaria, tropical diseases, and waterborne diseases; ensuring universal access to sexual and reproductive health services; as well as consolidating universal health coverage, grounded in access to essential, quality, safe, and affordable services and supplies for all^1^.

With regard to eliminating epidemics aligned with the commitments of the 2030 Agenda - whose diseases are socially determined and deepen historical inequalities - it is noteworthy that Sub-Saharan Africa accounts for more than 50% of people living with HIV/AIDS and TB. Seven countries in the region concentrate the highest number of cases (Botswana, Cameroon, Eswatini, Guinea, Guinea-Bissau, Malawi, and Zimbabwe); 10 countries face high burdens of TB and TB/HIV coinfection (Central African Republic, Congo, Ethiopia, Gabon, Kenya, Lesotho, Liberia, Namibia, Uganda, and Tanzania); five countries simultaneously concentrate high burdens of TB, TB/HIV coinfection, and multidrug-resistant TB (MDR/RR-TB) (Democratic Republic of the Congo, Mozambique, Nigeria, South Africa, and Zambia). Angola has a high burden of TB and MDR/RR-TB; Somalia and Zimbabwe stand out regarding MDR/RR-TB, with Somalia additionally leading the ranking in increasing TB incidence rates^2^.

In addition to this epidemiological reality, the region continues to be affected by cholera, such that, between January and July 2024, 14 countries reported 112,301 cases and 1,900 deaths due to lack of access to safe drinking water, poor housing conditions, lack of hygiene, and inadequate environmental sanitation^3^. Alongside this scenario, alarming rates of maternal and child mortality are also observed, contributing to a low human development index^4^ and deepening socio-regional adversities.

Other factors impacting the achievement of SDG 3 are related to armed conflicts and political instability that permeate Sub-Saharan Africa and significantly affect vulnerable populations^5^ ^-^ ^6^. Since 2021, seven countries in the region have experienced coups d’état: Chad (April 21, 2021), Mali (May 24, 2021), Guinea (September 5, 2021), Sudan (October 25, 2021), Burkina Faso (September 30, 2022), Niger (July 26, 2023), and Gabon (August 30, 2023). In addition to these coups, the region also faces the activity of terrorist groups, resulting in internal displacement and refugee flows, as seen in Mozambique, Nigeria, Kenya, Sudan, Mali, Democratic Republic of the Congo, Chad, Burkina Faso, Somalia, and Eritrea^5^. Further aggravating this scenario is the mismatch in public health service coverage in the region (below 60%), with significant disparities among countries^2^.

In light of these reflections, one questions - or rather, becomes indignant about - the real and (in)effective conditions for addressing the historical inequalities that permeate social and health production and reproduction in Sub-Saharan Africa, perpetuating the impoverishment of the region and the alarming impact of preventable, neglected, and communicable diseases, which intensify social injustices. On the other hand, it is necessary to recognize, albeit modestly, the efforts undertaken and progress achieved; however, the persistence of social problems, political instability, conflicts, and migration movements in the region project the commitments of the 2030 Agenda onto an increasingly unlikely horizon given the current context.

Bold policies and joint efforts to expand social protection and human rights in vulnerable territories are imperative for tackling the diseases affecting the region. This requires leadership and partnerships among governments, multilateral agencies, and civil society in the pursuit of sustainable funding and improvements in access to and quality of health services, as well as advances in policies aimed at the training, qualification, and retention of health human resources.

There is an urgent need for social mobilization in defense of the human dignity of the populations living in the region, with the overcoming of infrastructural and systemic deficits and the strengthening of the response capacity and resilience of health systems to address socially determined diseases, particularly communicable ones. Furthermore, from the perspective of access to quality services and supplies, the implementation of public policies ensuring the provision of safe drinking water and basic sanitation is indispensable, under the principles of human rights, equity, and the promotion of social justice.

Finally, reflecting on the challenges in pursuing universal access to health in Sub-Saharan Africa requires deep concern to understand antagonistic and synergistic social processes - historical and contemporary - marked by inequalities, diverse interests, and transnational asymmetries. In this scenario, progress toward the SDGs of the 2030 Agenda among African populations requires robust and coordinated social protection policies aligned with achieving a common goal: human well-being and dignity. Beyond geographical boundaries, existing borders must be transformed into links of solidarity and strategic cooperation in the region.
