# Supplementary material for: Sub-Saharan African Countries: what challenges for achieving universal access to health?
Source: Rev Lat Am Enfermagem. 2026 Jul 31;34:e5051. doi: 10.1590/1518-8345.0000.5051 (PMC13427257; doi:10.1590/1518-8345.0000.5051)
Supplement: Translation

# Translation: Países da África Subsaariana: quais desafios para o propósito do acesso à saúde para todos?

**DOI:** 10.1590/1518-8345.0000.5052

**Authors:** Fernando Mitano, Aline Aparecida Monroe, Pedro Fredemir Palha

**Affiliations:** 1 Universidade Lúrio, Faculdade de Ciências de Saúde, Nampula, NPL, Moçambique.; 2 Universidade de São Paulo, Escola de Enfermagem de Ribeirão Preto, Centro Colaborador da OPAS/OMS para o Desenvolvimento da Pesquisa em Enfermagem, Ribeirão Preto, SP, Brasil.

A saúde de qualidade para todos é almejada por países de todo mundo e isso requer esforços perenes, de forma singular e coletiva, de todos e, em especial, dos governos envolvidos. À luz dessas condições e do progresso das Nações Unidas com a propositura dos Objetivos de Desenvolvimento Sustentável (ODS) busca-se a superação de carências estruturais dos povos que vivem tanto nos países de baixa como de média e alta renda. O terceiro dos dezessete ODS preconiza, de forma imperativa, a garantia do acesso à saúde de qualidade e a promoção do bem-estar para todos os povos e em todas as fases da vida^1^. Espera-se que esse objetivo seja alcançando até 2030; contudo, diante das amplas e profundas iniquidades estruturais, tal projeção é, de fato, aplicável para os países da Africa Subsaariana?

Apesar dos esforços globais para o alcance desse objetivo, a África Subsaariana parece estar em descompasso com as demais regiões do mundo. Esta área, constituída por 47 países situados ao sul do Deserto de Saara (Figura 1), tem enfrentado diversos desafios de saúde, cuja mitigação exige compromisso político, gestão estratégica e intervenções coordenadas, alinhadas às metas do terceiro ODS. 

**Figura 1:** Países da África Subsaariana

Fonte: https://brasilescola.uol.com.br/geografia/as-duas-africas.htm

Tais metas preconizam a redução da taxa de mortalidade materna para menos de 70 por 100.000 nascidos vivos; a eliminação de mortes evitáveis de recém-nascidos; o fim das epidemias de tuberculose (TB), vírus da imunodeficiência adquirida (HIV), malária, doenças tropicais e aquelas transmitidas pela água; a garantia de acesso universal aos servicos de saúde sexual e reprodutiva, bem como a consolidação da universalidade na saúde, alicerçada no acesso a serviços e insumos essenciais, de qualidade, seguros e economicamente viáveis a todos^1^. 

No que diz respeito à eliminação de epidemias alinhadas aos compromissos da Agenda 2030, cujas doenças são socialmente determinadas e aprofundam iniquidades históricas, vale destacar que a África Subsaarina concentra mais de 50% das pessoas vivendo com HIV/aids e TB, sendo que sete países da região concentram maior número de casos (Botsuana, Camarões, Essuatíni, Guiné, Guiné-Bissau, Malawi e Zimbabwe); 10 países enfrentam altas cargas de TB e coinfecção TB/HIV (República Centro Africano, Congo, Etiópia, Gabão, Quênia, Lesoto, Libéria, Namíbia, Uganda, Tanzânia); cinco países concentram, concomitantemente, altas cargas de TB, coinfecção TB/HIV e TB multidrogarresistente (MDR/RR-TB) (República Democrática de Congo, Moçambique, Nigéria, África do Sul e Zâmbia). Angola possui elevada carga de TB e MDR/RR-TB; Somália e Zimbábue se destacam em relação à MDR/RR-TB, com a Somália liderando, adicionalmente, o *ranking* de aumento nas taxas de incidência da TB^2^.

Somada a essa realidade epidemiológica, a região continua a ser assolada pelo cólera, de modo que, no período de janeiro a julho de 2024, 14 países reportaram 112.301 casos e 1.900 óbitos em decorrência da falta de acesso à água potável, más condições habitacionais, falta de higiene e inadequado saneamento do meio ambiente^3^. Aliado a esse cenário, observam-se ainda índices alarmantes de mortalidade materna e infantil, os quais contribuem para o baixo índice de desenvolvimento humano^4^ e aprofundam as adversidades sociorregionais. 

Outros fatores que impactam o alcance do terceiro ODS estāo relacionados aos conflitos armados e instabilidades políticas que permeiam a Africa Subsaariana e afetam significativamente as populações vulnerabilizadas^5^ ^-^ ^6^. Desde 2021, sete países da região enfrentaram golpes de estado, a exemplo de Chade (21 de abril de 2021), Mali (24 de maio de 2021), Guiné (05 de setembro de 2021), Sudão (25 de outubro de 2021), Burkina Faso (30 de setembro de 2022), Níger (26 de julho de 2023) e Gabão (30 de agosto de 2023). Além de tais golpes, a região também enfrenta a atuação de grupos terroristas, que resultam em deslocamentos internos e fluxos de refugiados, como é o caso de Moçambique, Nigéria, Quênia, Sudão, Mali, República Democrática de Congo, Chade, Burkina Faso, Somália e Eritreia^5^. Agravando esse panorama, há que se mencionar o descompasso em relação à cobertura de serviços públicos de saúde na região (inferior a 60%), com importante disparidade entre os países^2^.

À luz das reflexões empreendidas, indaga-se, ou melhor, indigna-se em relação às reais e (in)efetivas condições de enfrentamento das iniquidades históricas que permeiam a produção e reprodução social e de saúde na África Subsaariana, perpetuando o empobrecimento da região e o alarmante impacto de doenças evitáveis, neglienciadas e transmissíveis, as quais acirram as injustiças sociais. Por outro lado, há que se reconhecer, ainda que timidamente, esforços envidados e avanços obtidos, contudo, a persistência de mazelas sociais, instabilidade política, confrontos e movimentos migratórios na região projetam os compromissos da Agenda 2030 em um horizonte cada vez mais improvável, diante da conjuntura analisada. 

Políticas arrojadas e esforços conjuntos para ampliar a proteção social e os direitos humanos em territórios vulnerabilizados são imperativos para o enfrentamento das doenças que assolam a região, exigindo protagonismo e parceriais governamentais, de agências multilaterais e sociedade civil na busca por financiamentos sustentáveis e incremento ao acesso e qualidade dos serviços de saúde, além de avanços na propositura de políticas voltadas à formação, qualificação e retenção de recursos humanos em saúde.

Urge a necessidade de mobilização social em defesa da dignidade humana dos povos que vivem na região, com superação de déficits infraestruturais, sistêmicos e o respectivo fortalecimento da capacidade de resposta e resiliência dos sistemas de saúde para o enfrentamento de doenças socialmente determinadas, com destaque para as transmissíveis. Ademais, na perspectiva de acesso a serviços e insumos de qualidade, faz-se indispensável a implementação de políticas públicas que assegurem a oferta de água potável e saneamento básico, sob a égide dos direitos humanos, equidade e promoção da justiça social. 

Por fim, refletir sobre desafios na busca pela universalidade do acesso à saúde na África Subsaariana exige uma profunda inquietude para compreender processos sociais antagônicos e sinérgicos, históricos e contemporâneos, marcados por iniquidades, interesses diversos e assimetrias transnacionais. Nesse cenário, o progresso dos ODS da Agenda 2030 na população africana requer políticas de proteção social robustas e coordenadas, alinhadas ao alcance de um mesmo objetivo comum, ou seja, o bem-estar e a dignidade humana. Para além de limites geográficos, há que se transpor as fronteiras existentes, convertendo-as em elos de solidariedade e cooperação estratégica na região. 

# Translation: Países del África Subsahariana: ¿qué desafíos para lograr el acceso universal a la salud?

**DOI:** 10.1590/1518-8345.0000.5050

**Authors:** Fernando Mitano, Aline Aparecida Monroe, Pedro Fredemir Palha

**Affiliations:** 1 Universidade Lúrio, Faculdade de Ciências de Saúde, Nampula, NPL, Moçambique.; 2 Universidade de São Paulo, Escola de Enfermagem de Ribeirão Preto, Centro Colaborador da OPAS/OMS para o Desenvolvimento da Pesquisa em Enfermagem, Ribeirão Preto, SP, Brasil.

La salud de calidad para todos es un objetivo deseado por países de todo el mundo, y requiere esfuerzos permanentes, tanto individuales como colectivos, de todos y, en especial, de los gobiernos involucrados. A la luz de estas condiciones y del progreso de las Naciones Unidas con la propuesta de los Objetivos de Desarrollo Sostenible (ODS), se busca superar las carencias estructurales de los pueblos que viven tanto en países de ingresos bajos como medios y altos. El tercero de los diecisiete ODS establece, de forma imperativa, garantizar el acceso a la salud de calidad y promover el bienestar para todos los pueblos en todas las etapas de la vida^1^. Se espera que este objetivo sea alcanzado para 2030; sin embargo, ante las amplias y profundas desigualdades estructurales, ¿es esta proyección realmente aplicable a los países del África Subsahariana?

A pesar de los esfuerzos globales para alcanzar este objetivo, el África Subsahariana parece estar en desventaja respecto a otras regiones del mundo. Esta área, compuesta por 47 países situados al sur del desierto del Sahara (Figura 1), ha enfrentado diversos desafíos sanitarios cuya mitigación exige compromiso político, gestión estratégica e intervenciones coordinadas, alineadas con las metas del ODS 3.

**Figura 1:** Países del África Subsahariana

Fuente: https://brasilescola.uol.com.br/geografia/as-duas-africas.htm

Estas metas incluyen reducir la tasa de mortalidad materna a menos de 70 por 100.000 nacidos vivos; eliminar las muertes evitables de recién nacidos; poner fin a las epidemias de tuberculosis (TB), virus de la inmunodeficiencia humana (VIH), malaria, enfermedades tropicales y aquellas transmitidas por el agua; garantizar el acceso universal a los servicios de salud sexual y reproductiva; así como consolidar la cobertura universal de salud, basada en el acceso a servicios e insumos esenciales, de calidad, seguros y económicamente viables para todos^1^.

En cuanto a la eliminación de epidemias alineadas con los compromisos de la Agenda 2030, cuyas enfermedades son socialmente determinadas y profundizan desigualdades históricas, cabe destacar que el África Subsahariana concentra más del 50% de las personas que viven con VIH/SIDA y TB. Siete países concentran el mayor número de casos (Botsuana, Camerún, Esuatini, Guinea, Guinea-Bisáu, Malawi y Zimbabue); 10 países enfrentan altas cargas de TB y coinfección TB/VIH (República Centroafricana, Congo, Etiopía, Gabón, Kenia, Lesoto, Liberia, Namibia, Uganda y Tanzania); cinco países concentran simultáneamente altas cargas de TB, coinfección TB/VIH y TB multirresistente (MDR/RR-TB) (República Democrática del Congo, Mozambique, Nigeria, Sudáfrica y Zambia). Angola presenta alta carga de TB y MDR/RR-TB; Somalia y Zimbabue se destacan en relación con la MDR/RR-TB, con Somalia liderando además el aumento en las tasas de incidencia de TB^2^.

Sumado a esta realidad epidemiológica, la región continúa siendo afectada por el cólera, de modo que, entre enero y julio de 2024, 14 países reportaron 112.301 casos y 1.900 muertes debido a la falta de acceso a agua potable, malas condiciones de vivienda, falta de higiene y saneamiento ambiental inadecuado^3^. A este escenario se suman alarmantes tasas de mortalidad materna e infantil, que contribuyen a un bajo índice de desarrollo humano^4^ y profundizan las adversidades sociorregionales.

Otros factores que impactan el logro del ODS 3 están relacionados con conflictos armados e inestabilidad política que afectan significativamente a las poblaciones vulnerables^5^ ^-^ ^6^. Desde 2021, siete países han experimentado golpes de Estado: Chad (21 de abril de 2021), Mali (24 de mayo de 2021), Guinea (05 de septiembre de 2021), Sudán (25 de octubre de 2021), Burkina Faso (30 de septiembre de 2022), Níger (26 de julio de 2023) y Gabón (30 de agosto de 2023). Además, la región enfrenta la acción de grupos terroristas que generan desplazamientos internos y flujos de refugiados en países como Mozambique, Nigeria, Kenia, Sudán, Mali, República Democrática del Congo, Chad, Burkina Faso, Somalia y Eritrea^5^. Este panorama se agrava por la baja cobertura de servicios públicos de salud en la región (inferior al 60%), con importantes disparidades entre países^2^.

A la luz de estas reflexiones, surge la indignación frente a las reales y (in)efectivas condiciones para enfrentar las desigualdades históricas que afectan la producción y reproducción social y sanitaria en el África Subsahariana, perpetuando el empobrecimiento de la región y el impacto alarmante de enfermedades prevenibles, desatendidas y transmisibles, que intensifican las injusticias sociales. Por otro lado, es necesario reconocer, aunque tímidamente, los esfuerzos realizados y los avances obtenidos; sin embargo, la persistencia de problemas sociales, la inestabilidad política, los conflictos y los movimientos migratorios proyectan los compromisos de la Agenda 2030 hacia un horizonte cada vez más improbable.

Se requieren políticas audaces y esfuerzos conjuntos para ampliar la protección social y los derechos humanos en territorios vulnerables. Esto exige liderazgo y alianzas entre gobiernos, agencias multilaterales y la sociedad civil para garantizar financiamiento sostenible, mejorar el acceso y la calidad de los servicios de salud, y avanzar en políticas de formación, cualificación y retención del personal sanitario.

Es urgente la movilización social en defensa de la dignidad humana de los pueblos que viven en la región, superando déficits estructurales y fortaleciendo la capacidad de respuesta y resiliencia de los sistemas de salud frente a enfermedades socialmente determinadas, especialmente las transmisibles. Asimismo, es indispensable implementar políticas públicas que aseguren el acceso al agua potable y al saneamiento básico, bajo los principios de derechos humanos, equidad y justicia social.

Finalmente, reflexionar sobre los desafíos para alcanzar la universalidad del acceso a la salud en el África Subsahariana exige comprender procesos sociales complejos, históricos y contemporáneos, marcados por desigualdades, intereses diversos y asimetrías transnacionales. En este contexto, el progreso de los ODS de la Agenda 2030 requiere políticas de protección social sólidas y coordinadas, orientadas a un objetivo común: el bienestar y la dignidad humana. Más allá de las fronteras geográficas, es necesario transformarlas en vínculos de solidaridad y cooperación estratégica en la región.
